# Optimizing seawater temperature conditions to increase the productivity of *ex situ* coral nurseries

**DOI:** 10.7717/peerj.13017

**Published:** 2022-03-09

**Authors:** Dakotah E. Merck, Chelsea G. Petrik, Alicia A. Manfroy, Erinn M. Muller

**Affiliations:** Mote Marine Laboratory, Summerland Key, Florida, United States

**Keywords:** Temperature, Coral, *Ex-situ*, Nursery, Growth

## Abstract

Large scale *ex situ* propagation of coral colonies for reef restoration is a relatively new and developing field. One of the many advantages of utilizing *ex situ* coral nurseries is the ability to optimize water quality conditions for coral health and survival. Slight alterations in environmental parameters (light, pH, temperature etc.) can affect the health and grow-out time of cultured coral, ultimately influencing production rates. However, corals are also subjected to pests associated with culture facilities such as ciliates, cyanobacterial blooms, and infectious diseases. Therefore, adjusting environmental parameters to optimize coral growth for a shorter *ex situ* residency time will lead to greater survival and faster restoration. Studies indicate that some coral species demonstrate parabolic tissue growth in response to increasing sea-surface temperatures until the maximum temperature tolerance is reached, whereafter they bleach. To maximize coral growth in Mote Marine Laboratory’s *ex situ* system, we tested the effect of two water temperature treatments (high temperature: 29.5 ± 0.03 °C; control: 25.2 ± 0.08 °C) on two coral species commonly used in reef restoration. To quantify this, we used four replicates of three genotypes each of *Montastraea cavernosa* (*n* = 12) and *Acropora palmata* (*n* = 12). Two-dimensional tissue area was recorded monthly using ImageJ and survival rates within each treatment were documented for 7 months. Results found that *M. cavernosa* had greater growth rates and equal survivorship in the high temperature treatment compared to the control treatment. *A. palmata* grew faster and had equal survivorship in the control treatment compared with the high temperature treatment. These results suggest that temperature preferences exist among coral species within *ex situ* systems and restoration practitioners should consider species-specific temperature regimes to maximize *ex situ* coral growth rates. This information is critical for optimizing production when corals are in the grow-out stage and should also be considered when designing *ex situ* systems to ensure temperature regulation can be controlled on a species-specific basis.

## Introduction

Coral reef ecosystems have undergone an extraordinary depletion of habitat-forming hard corals in recent years (Bruno & Selig, 2007; De’ath et al., 2012; Gardner et al., 2003; Jackson et al., 2014; Wilkinson, 2008). While systems like coral reefs possess the ability for gradual self-recovery (Connell, 1997; Gouezo et al., 2019), the frequency of extreme weather events and mass coral bleaching is increasing (Cheal et al., 2017; Hughes et al., 2018), diminishing the time and ability for recovery between such damaging events. While marine conservation has typically focused on passive habitat protection, the popularity of more assertive restoration practices has been increasing in recent years (Boström-Einarsson et al., 2020).

Innovations in active restoration practices have led to an increased number of large, high production *ex-situ* nurseries. Microfragmentation, utilizes a coral’s ability to multiply asexually by cutting or microfragmenting one large piece of coral into several smaller pieces (Forsman et al., 2015; Page & Vaughan, 2014). These fragmented pieces or “genetic clones” of the original coral are grown out on ceramic disks or ‘plugs’ in *ex situ*/land based systems in a practice known as coral gardening (Forsman et al., 2015; Page & Vaughan, 2014). When the corals reach 7 cm^2^, they are large enough to be microfragmented or outplanted on the reef. Those fragments selected for outplanting are secured to the reef substrate in arrays consisting of 5–10 clonal fragments spaced 1–2 cm apart in a circular formation. These corals then grow out over the substrate and, after touching, will fuse together to form a large colony approximately 20 cm in diameter, a process known as reskinning (Forsman et al., 2015). While wild colonies may take decades to reach sexual maturity, reskinning can create reproductively viable coral colonies within the span of a few years (Koch, Muller & Crosby, 2021). As the processes of microfragmentation, coral gardening, and reskinning are refined, the restoration field has embraced land based or *ex situ* nurseries. Compared to *in situ* field based coral nurseries, *ex situ* nurseries are easily accessed, a significant resource for education and outreach, and can efficiently produce large volumes of coral biomass. Due to these advantages, *ex situ* nurseries are quickly becoming the structure of choice for many restoration focused governmental and local organizations. *Ex situ* propagation of corals allows the restoration practitioner to control and manipulate many environmental conditions within the rearing system. Slight alterations in environmental parameters (light, pH, temperature etc.) can affect the health and grow-out time of cultured coral, ultimately influencing production rates (Jokiel & Coles, 1977; Coles & Jokiel, 1978).

The process of rearing coral on land from a fragmented or immature state can be time consuming depending on the species propagated. For example, when growing stony corals such as *Montastraea cavernosa*, the grow-out period from a freshly cut fragment of 1 cm^2^ in diameter microfragment to an “outplantable size” of 7 cm^2^ often takes up to 12 months. In comparison, acroporids, like *A. palmata*, grow much faster than boulder corals and can reach 7 cm^2^ in 6 months (Merck personal observation). It is during this varying grow-out period that opportunities arise for biological stressors to negatively affect the coral (Barton et al., 2020; Cassidy, 2009; Sweet, Jones & Bythell, 2011; Sweet et al., 2013). These stressors can include algal and cyanobacterial blooms that outgrow/smother coral (Cassidy, 2009), as well as outbreaks of marine pests, such as *Aiptasia* and hydrozoans. These stressors often cause tissue damage, thereby stunting the growth of *ex situ* corals. This slower growth rate increases the residency time that coral must stay within an *ex situ* system, producing a potential feedback loop of problems and delaying the ultimate goal of outplanting. Therefore, adjusting environmental parameters to optimize coral growth for a shorter *ex situ* residency time will lead to greater survival, higher turnover, faster biomass production, and lower cost per coral for reef restoration. Field studies indicate that some coral species demonstrate increased tissue growth in response to increasing sea-surface temperatures until the maximum temperature tolerance is reached, whereafter they reduce growth and potentially bleach (Coles & Jokiel, 1978; Jokiel & Coles, 1977; Miller, 1995). While this phenomenon is recognized in field settings, studies regarding how temperature affects Caribbean coral species in an *ex situ* nursery have yet to be performed. The present study examined both survivorship and two-dimensional tissue growth in two coral species of importance for restoration, *Montastraea cavernosa* and *Acropora palmata*, under two temperature regimes over a 7 month study period.

## Methods

### *Ex situ* nursery conditions

The two species of Caribbean coral, *Montastraea cavernosa* and *Acropora palmata*, used in the present study were sourced from the *ex situ* nursery at Mote Marine Laboratory’s International Center for Coral Reef Research and Restoration in Summerland Key, Florida (24°39′41″N, 81°27′16″W). Two of the three *M. cavernosa* genets (MC1 and MC11) were originally sourced from NOAA’s Key West Coral Rescue nursery in ~2014. The Rescue Nursery had acquired many corals associated with construction projects within Key West, particularly from local pier expansion projects. The third *M. cavernosa* genet, MC36, was sourced in 2019 from Higgs Heads, a reef also near Key West. The *A. palmata* were settled as sexual recruits at Mote and have since been reared in the land-based nursery. Two genotypes, AP5 and AP24, were settled in 2013 from a batch cross of gametes collected from three Upper Florida Keys reefs (Elbow, Horseshoe, and Sand Island). The remaining *A. palmata* genet, AP24, was settled in 2014 from a batch cross of gametes collected only from Elbow reef. For the present experiment, the corals were held for 8 months in total (October 20, 2020 to June 20, 2021), the first month being an acclimation period. Coral growth rate was documented monthly (first growth recording on October 20, 2020, and last growth recording on June 20, 2021) and survival was recorded during the following 7 months. Corals were held in an outdoor wet lab space under a 70% light reduction overhead shade structure supplied with particulate filtered, UV sterilized, and aerated near-shore and saltwater well derived seawater. Within this facility, corals were housed across two shallow flow-through style raceways (20 gallons; 4′1 Length × 1′10 Width × 1′1 Depth) with a flow rate of 43 cm^3^ s^−1^. Each raceway had a transparent plastic corrugated lid, which was placed on top of the open raceway in the event of inclement weather. One raceway was provided with control temperature seawater (target 25 °C) and one was maintained at high temperature conditions (target 29 °C) with a submersible aquarium heater (120 V titanium rod heater, Hygger). To ensure target temperature conditions were met across the 7 month grow-out period, water quality parameters including temperature, dissolved oxygen, salinity, and pH were measured twice daily (approx. 0800 and 1,500 GMT -5). Light levels were taken with an underwater quantum meter (MQ-210; Apogee Instruments Inc., Logan, UT, USA) at 10″ depth within an adjacent raceway approximately every 2 days at 1,200 (GMT -5). Light levels (maximum) were on average 316 ± 22 µmol m^2^ s^−1^ across the duration of this study.

### Temperature experiment

Each species used in this study consisted of three unique genotypes with four ramets per genotype produced *via* microfragmentation (Forsman et al., 2015; Page & Vaughan, 2014) for each temperature condition (*i.e*., control or high). In an effort to make the starting point of growth consistent amongst all genetic clones, the initial size for each rectangular fragment was 1.5–2 cm in diameter. Individuals were fixed to ceramic plugs (1¼″ Diameter, Boston Aqua Farms Inc., Windham, NH, USA) with cyanoacrylate gel (Bulk Reef Supply, Golden Valley, MN, USA). After fragmentation on 8 October 2020, corals were given time to heal. It should be noted that *A. palmata* fragments had an encrusting morphology and did not have apical polyps throughout this experiment. On 20 November 2020, corals were placed into either the control temperature tank (*n* = 24) or the high temperature tank (*n* = 24). Corals were given 1 month acclimation to their respective acclimation temperatures after fragmentation before the first (T0) tissue surface area data were collected.

### Surface area image analysis

Total tissue surface area was calculated for all replicates by taking overhead photographs using a digital camera (Olympus TG6). Two sets of photographs were taken with a ruler adjacent to the corals for scale, the first on 11/20/20, at the end of the acclimation period, and the last on 6/20/21, which was the end of the experiment. Each coral replicate was measured for respective 2D surface area (cm^2^) using ImageJ software (National Institute of Health, Bethesda, MD, USA) (Papke et al., 2021). Relative percent change in size was calculated from the difference in size at the end time point compared with the size at the initial time point divided by the initial size. Instances of mortality were recorded monthly among both temperature conditions. A binomial distribution was assigned to each coral replicate at each time point; 0 was given if the coral replicate was alive and 1 was given if the coral replicate was dead. The experiment was concluded after 7 months of temperature exposure.

## Results

### Temperature conditions

**Figure 1:**
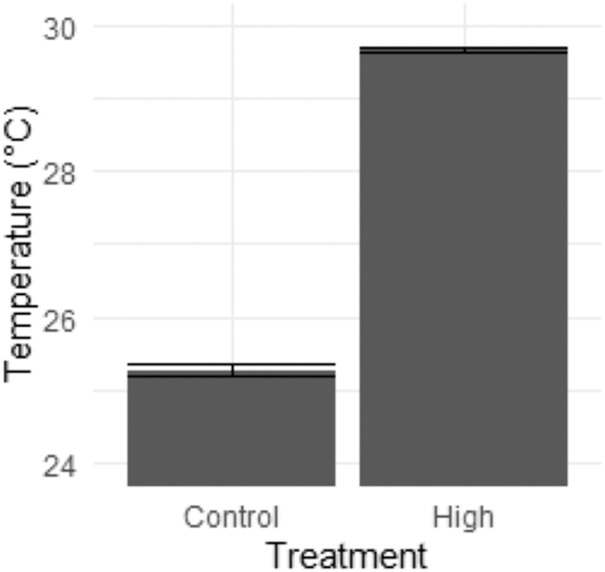
Temperature conditions. Mean (±SE) temperature of the control and high temperature raceways (right).

From 20 November 2020 to 20 June 2021, the control temperature raceway was maintained at an average daily temperature of 25.2 ± 0.08 °C (Fig. 1). The high temperature raceway was maintained at an average 29.5 ± 0.03 °C, where the temperatures were stabilized by submersible heaters (Fig. 1).

### Survivorship

Among the 24 fragments of *A. palmata*, survival probability remained high at 100% when held at both control and high temperatures during the 7 month grow-out period. There was no role of temperature conditions on survival for *A. palmata* fragments (Likelihood ratio test, Pr(*χ*2) = 1). Similarly, *M. cavernosa* survival probability was high at 100%, among the 24 fragments, across the grow-out period within both temperatures. There was no role of temperature on survival probability for *M. cavernosa* (Likelihood ratio test, Pr(*χ*2) = 1). *A. palmata* and *M. cavernosa* had similar survival probabilities across the 7 month grow-out period (Likelihood ratio test, Pr(*χ*2) = 1).

### Growth rates

**Figure 2:**
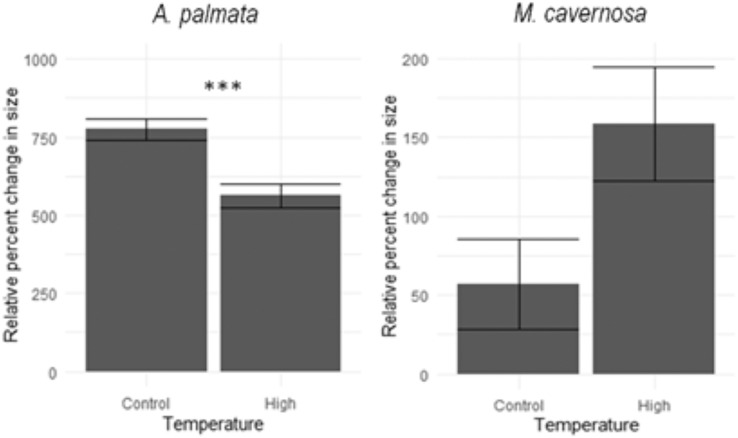
Species response-relative percent change in size. Average (±SE) relative percent change in size of A. *palmata* (left) and M. *cavernosa* (right) fragments after a 7 month period under control or high temperatures. Results from a *post hoc* test are presented as asterisks denoting significant differences among temperature treatments; *** denotes *p* < 0.001.

*A. palmata* fragments grew significantly more than *M. cavernosa* during the 7 month grow-out period, regardless of temperature treatment (ANOVA, F = 265.8, *p*-value < 0.001). *A. palmata* fragments held at control and high temperatures had on average a 776 ± 38% and 562 ± 34% increase in surface area, respectively (Fig. 2). The fragments within the control temperatures had a significantly higher relative percent change in surface area than the coral fragments held at high temperatures (ANOVA, F = 15.52, *p*-value < 0.001). *M. cavernosa* had on average a 57 ± 29% increase in surface area in the control temperature treatment and a 159 ± 36% increase in surface area in the high temperature treatment (Fig. 2). However, there was no significant difference in the relative percent change in size between *M. cavernosa* fragments held at control temperatures and the high temperature conditions (ANOVA, F = 33.8, *p*-value = 0.172).

### Genotype response

**Figure 3:**
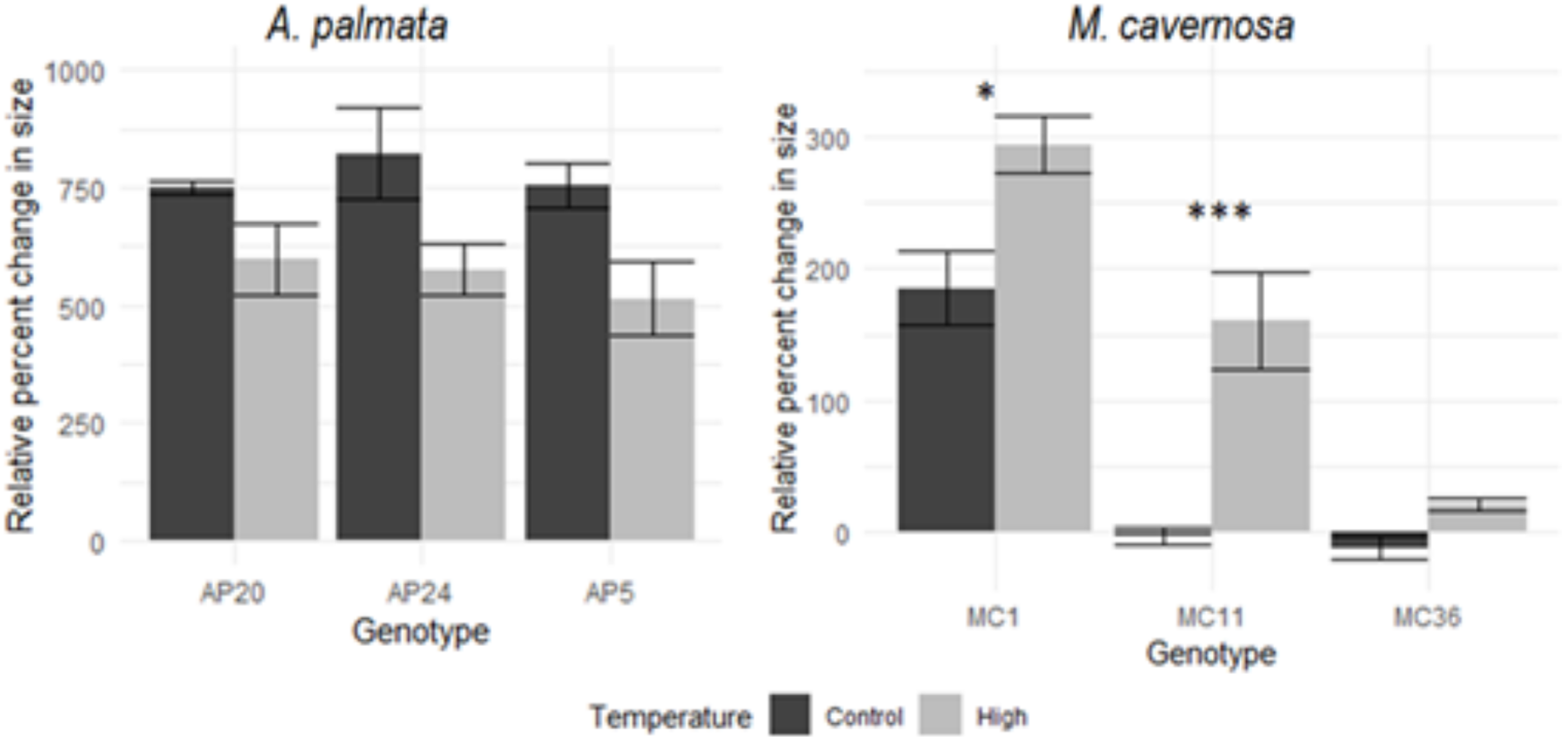
Genotype response-relative percent change in size. Average (±SE) relative percent change in size of three genotypes (AP5, AP20, AP24) of A. *palmata* (left) and three genotypes (MC1, MC11, MC36) of M. *cavernosa* (right) fragments after 7 month period in either control (black) or High (grey) temperatures. Results from a *post hoc* test are presented as asterisks denoting significant differences among temperature treatments; * denotes *p* < 0.05 and *** denotes *p* < 0.01.

*A. palmata* genotypes (AP5, AP20, AP24) had similar responses to temperature conditions, with higher growth for all genotypes (+755%, +822%, and +750%, respectively) in control temperatures compared with the same genets in the higher temperatures (+514%, +597%, +575%, respectively). Indeed, there were no significant differences of percent change in size detected among the genotypes of *A. palmata* (ANOVA, F = 0.473, *p* = 0.631). *M. cavernosa* fragments, however, had differing responses to control and high temperatures depending on genotype (MC1, MC11, MC36) (Fig. 3). There was a significant difference in growth rates among genotypes for *M. cavernosa* (ANOVA, F = 62.554, *p* < 0.001). The percent change in size when held at high temperatures were significantly higher than at control temperatures in genotype MC1 (Tukey *post hoc* test, *p*-value = 0.021) and MC11 (Tukey *post hoc* test, *p*-value < 0.001). The temperature treatment did not have an effect on percent change in size in genotype MC36 (ANOVA, *p*-value = 0.874).

## Discussion/conclusion

The present study demonstrated that survivorship was high in both species throughout the experiment under the two temperature regimes (25 °C and 29 °C). Indeed, all six genotypes used in this study (three *A. palmata* and three *M. cavernosa*) had 100% survivorship over the 7 month time period. Although no mortality was observed, if temperatures higher than the present study were used (>29.5 °C) there may have been mortality rates observed as this temperature threshold often induces symbiont expulsion (*i.e.*, heat stress) (Coles & Jokiel, 1978; Jokiel & Coles, 1977; Miller, 1995) especially in *A. palmata* (Williams et al., 2017). As neither species experienced mortality from the control or heated conditions, it is likely that the lower and upper thermal thresholds of these specific genotypes used from nursery grown corals fall outside the temperature ranges experienced within the present study. Indeed, Williams et al. (2017) showed *A. palmata* bleached after temperatures exceeded 30.5 °C within the Florida Keys indicating a critical temperature threshold associated with this branching coral species.

While temperature did not affect survival, the present study demonstrated that growth rates differed when corals were reared under different temperature conditions, a response that was species-specific. *Acropora palmata* grew optimally at 25.2 °C compared with 29.5 °C, whereas *M. cavernosa* grew more under the high temperature conditions compared with the control treatment. Previous studies showed corals often grow faster during summertime when temperatures are warmer and show reduced growth during winter months (Bak, Nieuwland & Meesters, 2009), suggesting corals held at warmer temperatures would stimulate growth. However, this response was not observed for *A. palmata* in the present study. The reduced growth rates of *A. palmata* under high temperature conditions may suggest the temperatures were reaching the thermal limit of this species, but without eliciting visual signs of stress. *A. palmata* is known to bleach at temperatures only slightly above the high temperature treatment conditions of the present study (Williams et al., 2017). Therefore, extensive exposure to 29.5 °C may have exceeded the physiological threshold of this more temperature sensitive species thus resulting in slower growth under high temperatures.

Alternatively, the symbiont type found within each species could have influenced the growth in response to the temperature conditions. *A. palmata* within Mote’s land-based nursery largely harbors algal symbionts of the genus *Durusdinium* (Fig. S1, Part A). These symbionts are known to be thermally tolerant, but may behave opportunistically and lead to reduced coral growth rates (Jones & Berkelmans, 2010; Jones & Berkelmans, 2011; Little, van Oppen & Willis, 2004). However, Cunning et al. (2015) showed the negative effects of *Durusdinium* communities on coral growth rates were diminished at higher temperatures with no significant differences in growth rate identified between *Pocillopora damicornis* harboring *Cladocopium* and *Durusdinium* at 29 °C. The results of the present study, however, showed that the *A. palmata*, which all harbor *Durusdinium*, had reduced growth under high temperatures. Therefore, if the algal symbiont is influencing growth rates then *Durusdinium* may be functioning differently within *A. palmata* compared with *P. damicornis*. Acropora *palmata* corals within the Florida Keys are not known to harbor *Durusdinium* symbionts and the local association is often with *Symbiodinium* ‘*fitti*’ (Durante et al., 2019). Therefore, this novel host-symbiont association may lead to novel phenotypes of the coral holobiont and more research is needed to fully understand the extent of this association.

Interestingly, the results of the present experiment showed that *M. cavernosa* had more variable growth rates among genotypes compared with *A. palmata*, which showed a similar response regardless of genotype. Throughout the experiment two out of three *M. cavernosa* genotypes had higher growth rates when held at high temperature conditions than when held at control temperatures. Other studies found similar results where *M. cavernosa* showed faster growth rates under warm water conditions (Manzello et al., 2015). The present study revealed that optimal thermal regimes vary by genotype in *M. cavernosa* (Drury et al., 2017). Two of three *M. cavernosa* genotypes, MC1 and MC11, in which we have symbiont typing confirmed harbored exclusively *Cladocopium* (Fig. S1, Part B). Data on algal symbiont diversity were not available for genotype MC36 at the time of this study, however, the lack of diversity present within the algal symbiont community of several other *M. cavernosa* genets from Mote’s land-based nursery suggest the common garden environment of the land-based nursery homogenizes the algal symbiont community. As symbiont profile data were not available for genotype MC36, it is possible that the variability in thermal response of this genotype may be attributable to a different algal symbiont composition. As all other screened genotypes of *M. cavernosa* possessed high abundances of only a few strains of the genus *Cladocopium*, we believe that it is unlikely that genotype MC36 has a different algal symbiont profile as this genotype was co-housed with the other screened genotypes. Future experiments should assess why genotypic variance in growth rate was more prevalent in *M. cavernosa* than *A. palmata* and if this trend holds true when compared to additional genotypes and other species.

E*x situ* nurseries are constructed differently and have different water sources with different seawater compositions and properties often based on location, funding sources, and institutional preferences. Some *ex situ* nurseries are operated indoors with recirculating systems supplied with stored seawater reserves. The entirety of the present study, however, was conducted in a flow-through outdoor wet lab in order to replicate the conditions that Mote Marine Laboratory’s *ex situ* corals are subjected to on a daily basis (sunlight, wind, weather etc.). Considering specific characteristics associated with every land-based nursery may influence how each restoration organization plans for the propagation of specific coral species. Many organizations, however, may assume that maintaining a consistent temperature regime throughout an entire *ex situ* system that simply does not exceed temperature thresholds is all that is necessary for growing corals. The present study suggests that land-based nurseries should optimize water temperatures that are species specific. Integrating this information into planning a land-based nursery at the time of creation could reduce costs and ultimately optimize performance and nursery production. While coral nurseries are often used to reduce biological and environmental fluctuations, there is the issue of land-based conditions leading to coral stress or mortality (*i.e.*, pests, algal blooms, and pathogens) making long term coral rearing quite labor intensive and challenging. However, personal observation by D. Merck indicates that the high temperature treatment, which accelerated growth in *M. cavernosa*, did not notably worsen the prevalence of marine pests or algal blooms. However, harmful algal and cyanobacterial growth was observed to bloom in the control tank, where the *A. palmata* grew the best, *vs* the heated tank towards the end of study. By identifying optimal temperatures within tanks that enhance corals’ abilities to grow while also reducing pressures from algae and pests in the tanks ultimately produces ideal rearing conditions. Finding these optimal conditions for each coral species may be regionally specific, but may also be a critical component for optimizing production of corals for restoration.

Many future studies can be conducted to identify the ideal conditions for an *ex situ* nursery using the present study as a preliminary basis. Improvements to the present study should also be considered. For example, larger replication and greater genetic diversity could further identify the ideal conditions that induce the fastest growth rate for *ex situ* grown corals. This additional replication could also create the opportunity to better quantify the dynamics of genotypic expression on growth rate and determine whether optimal temperatures are consistent for each species among different regions within its range. Alternatively, distinguishing temperature preference amongst similarly related species, such as comparing *Acropora cervicornis* with *A. palmata* and *Orbicella faveolata* with *M. cavernosa*, could supply more information and expand the knowledge associated with the temperature preferences of more closely or more disparate coral species. Furthermore, utilizing technologies such as 3D modeling can produce a more inclusive metric of size over time (Koch et al., 2021) and measuring calcification rates through the change in buoyant weight technique could be a more encompassing metric over a long-term study.

In conclusion, temperature preferences that produce optimal growth are present in both *A. palmata* and *M. cavernosa* in an *ex situ* system, however, the two species showed different responses associated with the treatments. Finding optimal temperatures to grow-out corals within *ex situ* nurseries is essential for restoration practitioners to maintain coral health and increase efficiency. Small scale experiments like the one presented here can be conducted at low cost and provide critical information for land-based coral nursery managers and practitioners. These results ultimately aid in optimizing the production of corals within land-based nurseries, reducing costs associated with restoration efforts, and increasing the efficiency of the methodologies. Although restoration is happening at an accelerated pace, the continued degradation of reefs due to long-standing issues such as climate change and disease outbreaks suggests every effort is essential to preserve some semblance of coral reef ecosystems, especially in places such as Florida’s Coral Reef.

## Supplemental Information

10.7717/peerj.13017/supp-1 Supplemental Information 1 Growth rate figures.

10.7717/peerj.13017/supp-2 Supplemental Information 2 Code for survival analysis.

10.7717/peerj.13017/supp-3 Supplemental Information 3 Growth rate raw data.

10.7717/peerj.13017/supp-4 Supplemental Information 4 Code for temperature figures.

10.7717/peerj.13017/supp-5 Supplemental Information 5 Code for growth rate analysis of variance.

10.7717/peerj.13017/supp-6 Supplemental Information 6 Growth rates species figure.

10.7717/peerj.13017/supp-7 Supplemental Information 7 Code for growth rates by genotype figures.

10.7717/peerj.13017/supp-8 Supplemental Information 8 Survival analysis dataset.

10.7717/peerj.13017/supp-9 Supplemental Information 9 Supplemental figures. Table#.(a) Two-way analysis of variance between species (*A. palmata* and *M. cavernosa*) and Temperature treatment (control or high) on the relative percent change in size. (b) Two-way analysis of variance between Temperature treatment (control or high) and Genotype (AP5, AP20, AP24) on the relative percent change in size. (c) Two-way analysis of variance between Temperature treatment (control or high) and Genotype (MC1, MC11, MC36) on the relative percent change in size. Bolded p values were significant (*p* < 0.05).

10.7717/peerj.13017/supp-10 Supplemental Information 10 Supplemental Methods and Figures for symbiosis.

10.7717/peerj.13017/supp-11 Supplemental Information 11 Temp log dataset.

10.7717/peerj.13017/supp-12 Supplemental Information 12 Temperature dataset for figures.

## Additional Information and Declarations

### Data Availability

The following information was supplied regarding data availability: Data and code to create Temperature, growth percent change in species and genotypes are available in the Supplemental Files.
