# Supplementary figures and images for: Optimizing seawater temperature conditions to increase the productivity of ex situ coral nurseries
Source: PeerJ. 2022 Mar 9;10:e13017. doi: 10.7717/peerj.13017 (PMC8917797; doi:10.7717/peerj.13017)

**
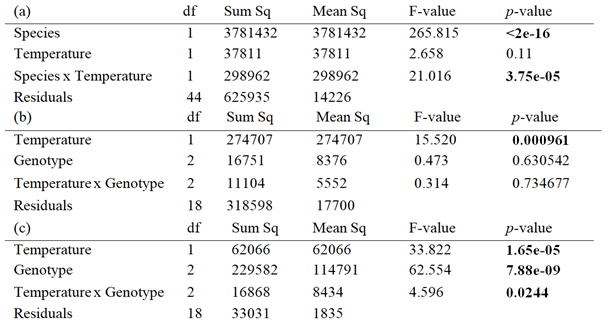
**

Supplement: Supplemental Information 9 — Table#.(a) Two-way analysis of variance between species (A. palmata and M. cavernosa) and Temperature treatment (control or high) on the relative percent change in size. (b) Two-way analysis of variance between Temperature treatment (control or high) and Genotype (AP5, AP20, AP24) on the relative percent change in size. (c) Two-way analysis of variance between Temperature treatment (control or high) and Genotype (MC1, MC11, MC36) on the relative percent change in size. Bolded p values were significant (p < 0.05). [file peerj-10-13017-s009.docx]
