# Supplementary material for: Optimizing seawater temperature conditions to increase the productivity of ex situ coral nurseries
Source: PeerJ. 2022 Mar 9;10:e13017. doi: 10.7717/peerj.13017 (PMC8917797; doi:10.7717/peerj.13017)
Supplement: Supplemental Information 10 [file peerj-10-13017-s010.docx]

Supplemental Methods and Figures

*Algal symbiont ITS profiling*

Ten unique genotypes of *Montastraea cavernosa* were sampled from Mote Marine Laboratory’s *ex situ* nursery in 2021. DNA was extracted from samples using the E.Z.N.A.® Soil DNA Kit (Omega Bio-Tek, Norcross, GA, USA) and manufacturer’s protocol. After quality and purity examination, the ITS2 region of the rDNA was amplified by PCR using primers (F: 5′GAATTGCAGAACTCCGTG-3′; R: 5′ GGATCCATATGCTTAAGTTCAGCGGGT-3′) designed to produce 330–360 bp ITS2 fragments (LaJeunesse and Trench, 2000). A polymerase chain reaction (PCR) was performed using the HotStarTaq Plus Master Mix Kit (Qiagen, USA) under the following conditions: 94°C for 5 minutes, followed by 30 cycles of 95°C for 30 seconds, 53°C for 40 seconds and 72°C for 1 minute, followed by a final elongation step at 72°C for 10 minutes. After amplification, PCR products were checked in 2% agarose gel to determine the success of amplification and the relative intensity of bands. Multiple samples were pooled based on DNA concentrations and were purified using calibrated Angencourt Ampure XP beads (Beckman Coutler, CA, USA). Then a pooled DNA library was generated using the Illumina TruSeq DNA library preparation protocol. Paired-end sequencing was performed at MR DNA (www.mrdnalab.com, Shallowater, TX, USA) using a single flow cell on a MiSeq following the manufacturer’s guidelines. Sequence data were demultiplexed, joined, sequences <150bp removed, and sequences with ambiguous base calls removed. Sequences were quality filtered using a maximum expected error threshold of 1.0 and dereplicated. Unique sequences were identified and chimeras were removed, thereby providing a denoised sequence or zOTU (zero-radius OTU). Final zOTUs were taxonomically classified using BLASTn against a curated database derived from NCBI ([www.ncbi.nlm.nih.gov](http://www.ncbi.nlm.nih.gov)). Relative proportions of reads mapping to the top 20 unique zOTUs were used to estimate symbiont diversity of each genotype.

*Algal symbiont 2bRAD profiling*

Fifty-three unique genotypes of *Acropora palmata* were sampled from Mote Marine Laboratory’s *ex situ* nursery in 2019. DNA was extracted from samples using the Qiagen DNeasy PowerSoil Pro Kit and manufacturer’s protocol. Illumina sequencing libraries were constructed by fragmenting DNA using a Type IIB restriction endonuclease, generally following Wang et al. (2012). Sequencing adapters were ligated to each fragment and sample-specific barcodes incorporated via PCR. The resulting libraries were quantified and pooled for sequencing on the Illumina 2500 at the University of Southern California. Resulting read data were demultiplexed and quality filtered using custom Perl scripts (<https://github.com/ckenkel/2bRAD_utilities>). High quality reads were mapped to a combination of the host reference genome (I. Baums/S. Kitchen pers. comm.) and representative symbiont transcriptomes for four Symbiodiniaceae genera (*Symbiodinium, Breviolum, Cladocopium,* and *Durusdinium*) following Manzello et al. (2019)(Aranda, M., et al.2016)(Huanle., et al 2017)( Shoguchi., et al 2013). The relative proportion of reads producing highly unique matches (with mapping quality 40 or better) to each reference genome was used to estimate symbiont diversity of each genotype.


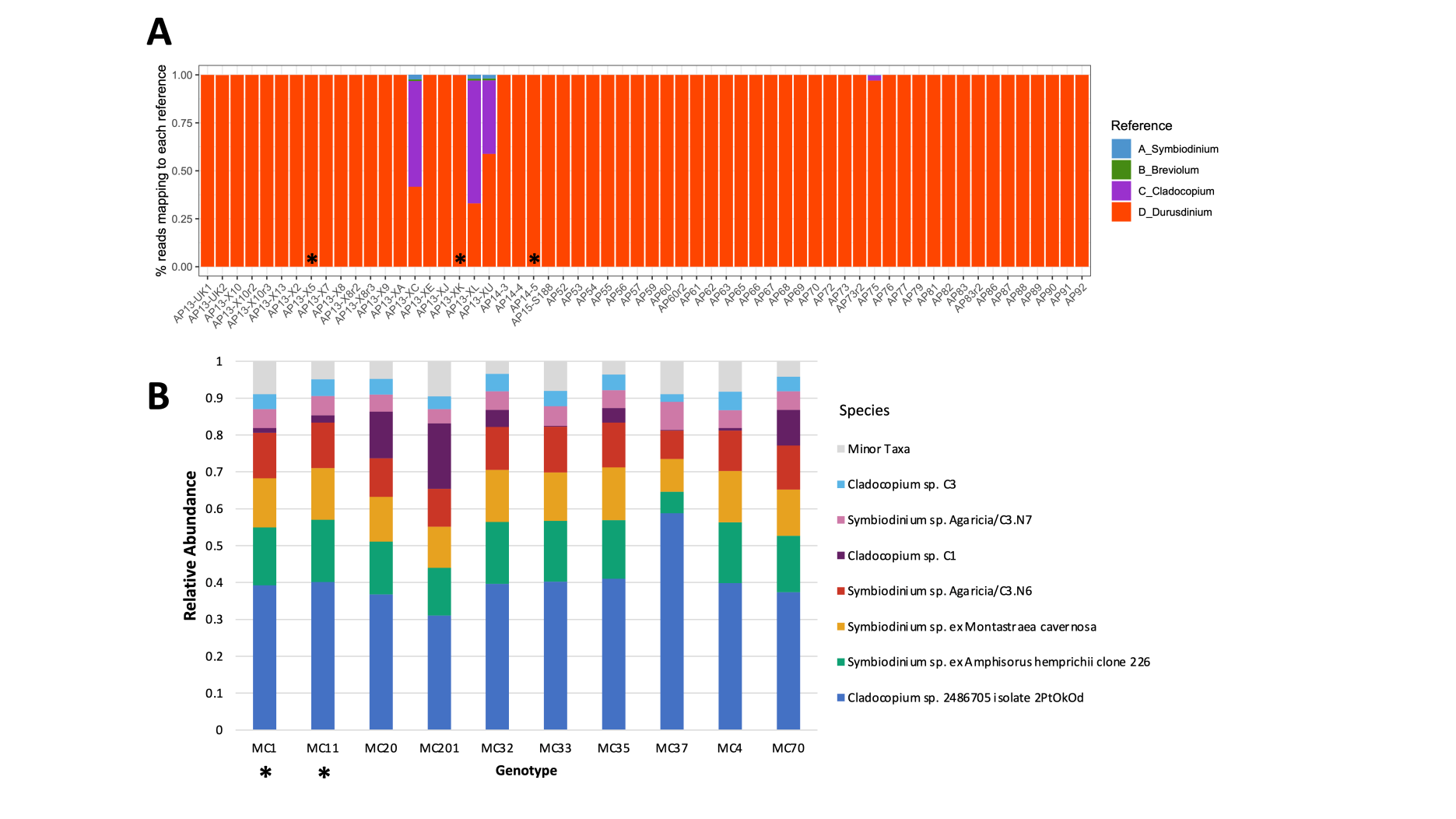


Supplemental Figure 1. Algal symbiont diversity of 53 genotypes of *A. palmata* (A) and ten genotypes of *M. cavernosa* (B) was assessed using 2bRAD sequencing and ITS2 amplicon sequencing, respectively. Of fifty-three genotypes of *A. palmata* sampled in 2019, 100% of reads were found to map to the reference genome of the genus *Durusdinium* in fifty genotypes, while three genotypes were found to also harbor the genus *Cladocopium* (Supplemental Figure 1). All three genotypes of *A. palmata* used in this study (AP5(AP13-X5), AP20(AP13-XK), AP24(AP14-5) were found to be dominated with *Durusdinium.* All genotypes assessed of *M. cavernosa* (sampled in 2021) were found to harbor low diversity of the family *Symbiodiniaceae*, with only seven unique strains dominating across all samples. All seven strains fell within the genus *Cladocopium.* Two of the three genotypes of *M. cavernosa* used in this study (MC1 and MC11) were included in this analysis. Data on algal symbiont diversity was not available for genotype MC36 at the time of this study.

Works cited

1. Wang, S., Meyer, E., McKay, J.K., and Matz, M.V. (2012). 2b-RAD: a simple and flexible method for genome-wide genotyping. Nat Methods *9*, 808–810.

2. Lajeunesse, T., and Trench, R. (2000). Biogeography of two species of Symbiodinium (Freudenthal) inhabiting the intertidal sea anemone Anthopleura elegantissima (Brandt). The Biological Bulletin *199*, 126–134.

3. Aranda, M., et al. “Genomes of Coral Dinoflagellate Symbionts Highlight Evolutionary Adaptations Conducive to a Symbiotic Lifestyle.” *Scientific Reports*, vol. 6, no. 1, 2016, https://doi.org/10.1038/srep39734.

4. Liu, Huanle, et al. “Symbiodinium Genomes Reveal Adaptive Evolution of Functions Related to Symbiosis.” 2017, https://doi.org/10.1101/198762.

5. Manzello, Derek P., et al. “Role of Host Genetics and Heat‐Tolerant Algal Symbionts in Sustaining Populations of the Endangered Coral Orbicella Faveolata in the Florida Keys with Ocean Warming.” *Global Change Biology*, vol. 25, no. 3, 2019, pp. 1016–1031., https://doi.org/10.1111/gcb.14545.

6. Shoguchi, Eiichi, et al. “Draft Assembly of the Symbiodinium Minutum Nuclear Genome Reveals Dinoflagellate Gene Structure.” *Current Biology*, vol. 23, no. 15, 2013, pp. 1399–1408., https://doi.org/10.1016/j.cub.2013.05.062.
